# Nine-Year Surveillance of Candida Bloodstream Infections in a Southern Italian Tertiary Hospital: Species Distribution, Antifungal Resistance, and Stewardship Implications

**DOI:** 10.3390/jpm16010017

**Published:** 2026-01-02

**Authors:** Anna Maria Spera, Veronica Folliero, Chiara D’Amore, Biagio Santella, Flora Salzano, Tiziana Ascione, Federica Dell’Annunziata, Enrica Serretiello, Gianluigi Franci, Pasquale Pagliano

**Affiliations:** 1 Infectious Disease Unit, University Hospital OORR San Giovanni di Dio e Ruggi d’Aragona, 84131 Salerno, Italy; aspera@unisa.it; 2 Department of Medicine, Surgery and Dentistry “Scuola Medica Salernitana”, University of Salerno, 84081 Baronissi, Italy; vfolliero@unisa.it (V.F.); bsantella@unisa.it (B.S.); flsalzano@unisa.it (F.S.); federica.dellannunziata@unicampania.ot (F.D.); enrica.serretiello87@hotmail.it (E.S.); gfranci@unisa.it (G.F.); 3 UOC Clinica Infettivologica AOU San Giovanni di Dio e Ruggi d’Aragona, 84131 Salerno, Italy; chiara.damore@sangiovannieruggi.it; 4 Service of Infectious Diseases, Cardarelli Hospital, 80131 Naples, Italy; tiziana.ascione@hotmail.it

**Keywords:** candidemia, antifungal resistance, species distribution, clinical settings, stewardship, surveillance

## Abstract

**Purpose**: Candida bloodstream infections remain a major global health challenge, with mortality rates approaching 40%. Beyond classical immunocompromised status, recent evidence highlights additional risk factors, including iatrogenic immunosuppression, advanced age, prolonged hospitalization, exposure to broad-spectrum antibiotics, and total parenteral nutrition. While *Candida albicans* (*C. albicans*) remains the most common species in Europe and the USA, non-*albicans* species, particularly *Nakaseomyces glabratus* (*N. glabratus*), *Candida tropicalis* (*C. tropicalis*), and *Candida parapsilosis* (*C. parapsilosis*), are emerging worldwide. **Methods**: This retrospective observational cohort study was conducted at the University Hospital “San Giovanni di Dio e Ruggi d’Aragona” in Salerno, Italy, from January 2015 to December 2024. It included all patients with at least one positive blood culture for *Candida* species. Demographic data, hospital ward of admission, and antifungal susceptibility profiles were collected and analyzed using SPSS software (IBM SPSS Statistics for Mac, version 30 (IBM Corp., Armonk, NY, USA)). **Results**: The incidence rate is 48.7 new isolates per one thousand patient-days, with a trend of increasing episodes over time among a total of 364 patients. Most cases occurred in medical wards (59.5%), where patients were older (median age 76 (17). *C. albicans* accounted for 57.9% of isolates, and a significant association was found between species distribution and hospital unit (*p* < 0.05). Resistance to fluconazole, voriconazole, and amphotericin B increased among *C. albicans*, with similar trends in *N. glabratus* and *C. parapsilosis*. **Conclusions**: This large single-center cohort highlights both the persistent dominance of *C. albicans* and the worrisome rise in resistance among *C. parapsilosis*. Given the aging patient population and increasing antifungal resistance, local epidemiological data are crucial to guide empirical therapy. Our findings underscore the need for multidisciplinary antifungal stewardship programs to optimize personalized treatment strategies and contain the emergence of resistant strains.

## 1. Lay Summary

Candida bloodstream infections are rising, especially in older hospitalized patients. *C. albicans* is still most common, but drug-resistant strains like *C. parapsilosis* are increasing. Local data and better antifungal strategies are key to improving treatment outcomes.

## 2. Introduction

Bloodstream infections (BSIs) rank among the top seven causes of death, with over two million cases reported annually, resulting in approximately 250,000 deaths and case fatality rates ranging from 13% to 20% [1]. Evidence on BSIs has focused primarily on bacterial pathogens, which are the most common causative agents. However, although less frequently detected, fungal BSIs also play a crucial role and are increasingly recognized for their clinical and epidemiological influence [2].

Among fungal BSIs, *Candida* spp. reports the highest incidence and can support life-threatening bloodstream infections, with a reported global average mortality rate of 40%, reaching even higher levels in critically ill patients. Although candidemia was historically associated with neutropenic and severely immunocompromised individuals, other relevant risk factors have now emerged. These include iatrogenic immunosuppression, aging, hospitalization in intensive care units (ICUs), total parenteral nutrition, administration of broad-spectrum antibiotics, and organ dysfunction. Regardless of the underlying causes, candidemia is strongly associated with prolonged hospitalization and has a significant impact on both healthcare costs and patient outcomes [3,4,5,6,7].

Understanding local epidemiology of Candida BSIs is essential for the effective management of these invasive fungal infections (IFIs) [8]. Indeed, the distribution of *Candida* species can be influenced by patient comorbidities, prior antifungal exposure, and local hospital-specific factors. While *C. albicans* remains the leading cause of candidemia, accounting for 38% to 54% of cases in Europe and the USA, epidemiological trends are shifting [9,10]. Cases of candidemia caused by *Candida* non-*albicans* (*C.* non-*albicans*) species, such as *Nakaseomyces glabratus* (*N. glabratus*) and *Candida tropicalis* (*C. tropicalis*), are rising in the USA and Asia, while *Candida parapsilosis* species complex (*C. parapsilosis*) is becoming more frequent in Europe [11,12].

Several antifungal strategies are currently used to manage infections caused by *Candida* spp., including polyenes (e.g., amphotericin B), azoles (e.g., fluconazole and voriconazole), and echinocandins (e.g., caspofungin, micafungin, anidulafungin) [13]. Polyenes exert fungicidal activity by binding to ergosterol and compromising membrane integrity, whereas azoles inhibit ergosterol biosynthesis, resulting in fungistatic effects [14]. Echinocandins, which inhibit β-(1,3)-D-glucan synthase, are considered first-line therapy for invasive candidiasis due to their potent activity, favorable safety profile, and limited cross-resistance [15]. Additional therapeutic options, including flucytosine or combination regimens, are used in selected clinical settings, particularly in severe or refractory infections. Emerging antifungal agents targeting novel pathways, such as fungal respiration or chitin synthesis, are currently under investigation and may provide alternative strategies soon [16].

Despite the availability of these therapeutic approaches, antifungal resistance among *Candida* species is a growing concern. Increasing reports of azole resistance have been attributed to overexpression of efflux pumps and alterations in ergosterol biosynthesis [17]. Even more critical is the increase in intrinsically less susceptible *C.* non-*albicans* species, such as *N. glabratus*, which often exhibits reduced azole susceptibility and is prone to developing echinocandin resistance [18]. Overall, these trends highlight the urgent need for improved anti-fungal stewardship to counteract the evolving resistance landscape. The primary objective of this study is to evaluate the epidemiology of BSIs caused by *Candida* in a University Hospital in Southern Italy. Specifically, we planned this study to identify the incidence of candidemia in different hospital settings, including medical, surgical, and ICUs. Additionally, we aim to investigate the distribution and antifungal susceptibility profiles of *Candida* species over nine years. The goal is to support updated data to develop targeted antifungal strategies.

## 3. Materials and Methods

### 3.1. Patient Inclusion

This retrospective observational cohort study was conducted between 1st January 2015 and 31st December 2024, at University Hospital “San Giovanni di Dio e Ruggi d’Aragona” in Salerno, Italy. Adult patients with at least one positive blood culture for *Candida* species were included. In cases where multiple isolates of different *Candida* species were obtained for a single patient, a new episode was defined as a positive blood culture occurring at least 14 days after the previous episode [19].

### 3.2. Inclusion Criteria

Inclusion criteria for the study were as follows: (i) admission to the San Giovanni di Dio and Ruggi d’Aragona University Hospital in Salerno, Italy; (ii) presence of at least one positive blood culture for *Candida* species; (iii) and clinical evidence of signs and symptoms consistent with a bloodstream infection.

### 3.3. Identification of Candida spp. and Antifungal Susceptibility Testing

Blood samples were collected via peripheral venipuncture or a central venous line and inoculated into two or three sets of aerobic and anaerobic blood culture bottles, with a standard volume of 8–10 mL per bottle for adults. Bottles were incubated using the Bact/ALERT system (bioMérieux, Marcy-l’Étoile, France). Positive aerobic cultures were subcultured onto Columbia CNA agar, MacConkey agar, chocolate agar, and Sabouraud dextrose agar and incubated at 37 °C for 24–48 h (bioMérieux, Marcy-l’Étoile, France). Colonies were identified to the species level using Matrix-Assisted Laser Desorption/Ionization Time-of-Flight (MALDI-TOF) mass spectrometry with the Vitek^®^ MS system (bioMérieux, Marcy-l’Étoile, France). Antifungal susceptibility testing was performed using the VITEK^®^ 2 system with the AST-YS08 card. The antifungal agents tested included amphotericin B, caspofungin, fluconazole, micafungin, and voriconazole. All procedures were conducted in accordance with the manufacturer’s instructions and standard microbiological protocols [20,21]. Antifungal susceptibility testing was performed exclusively using commercial methods (Vitek 2, AST-YS08 card) (bioMérieux, Craponne, France), without confirmation by reference broth microdilution according to CLSI or EUCAST standards. This methodological characteristic may affect the interpretability and comparability of MIC values across studies.

### 3.4. Data Analysis

Statistical analysis was conducted using the chi-square (χ^2^) test or Fisher’s exact test, as appropriate, to explore possible associations between categorical variables. Continuous variables have been reported as median and IQR, and the Mann–Whitney *U* test was used to assess the differences encountered. When significant associations were identified, post hoc analysis was conducted using adjusted standardized residuals to better understand which specific factors contributed to the observed differences. A *p*-value < 0.05 was significant. All analyses were conducted using IBM SPSS Statistics for Mac, version 30 (IBM Corp., Armonk, NY, USA).

### 3.5. Ethics Statement

The study was conducted with the approval of the institutional review board and in accordance with the principles outlined in the Declaration of Helsinki. Informed consent was obtained from all subjects involved in the study.

## 4. Results

### 4.1. Patient Characteristics

**Figure 1:**
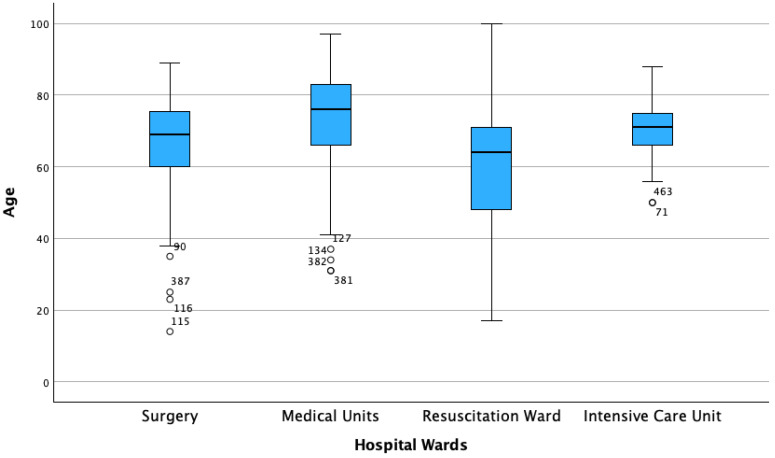
Box plots illustrating the median age and interquartile range (IQR) across hospitalization wards.

During the study period, 487 episodes (namely strains) of candidemia were identified from blood cultures of 364 patients. A rising trend in the number of cases was observed between 2015 and 2024. Specifically, the annual distribution of the cases was as follows: 27 cases were identified in 2015, 15 in 2016, 28 in 2017, 32 in 2018, 33 in 2019, 29 in 2020, 49 in 2021, 84 in 2022, 68 in 2023 and 72 in 2024. Most patients were male (265 cases, 54.4%). The median age was 71 (17) years. Male patients were younger than female patients (69 (18) vs. 75 (17)). (Table 1). Patients admitted to medical units were the oldest, with a median age of 76 (17) years, followed by those in ICU 71 (10) years, in surgical units (69 (18) years), and resuscitation unit (namely, Department of Anesthesiology and Critical Care or DACC) (64 (24) years), (Figure 1).

**Figure 2:**
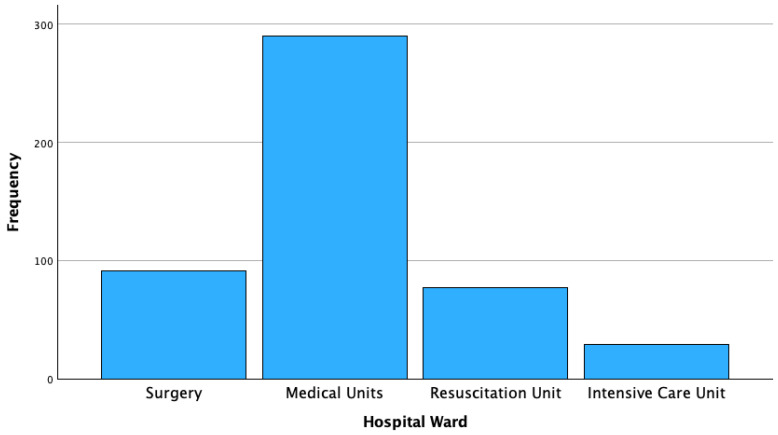
Frequency of Candida species isolated, stratified by hospital ward.

The majority of candidemia cases occurred in medical units (290/487 strains, 59.5%), followed by surgery units (91/487, 18.7%), DACC (77/487; 15.8%) and ICU (29/487; 6%). (Table 2, Figure 2).

### 4.2. Candida Species Distribution

**Figure 3:**
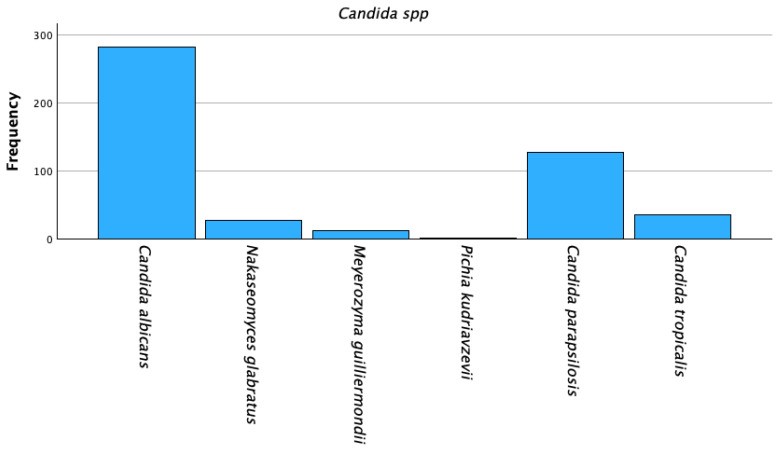
*Candida* spp. isolation on strains.

*C. albicans* was isolated in 282 specimens (57.9%), *Candida* non-*albicans* was found in 205 specimens (42.1%). Among *C.* non-*albicans* species, *C. parapsilosis* and *C. tropicalis* were the most frequent species, accounting for 128 (26.3%) and 35 (7.2%) isolates. (Table 3, Figure 3).

A statistically significant association was observed between the distribution of *Candida* species and the hospitalization unit. *C. albicans* was the predominant species isolated in the medical unit, as confirmed by the chi-square (χ^2^) test. (Table 4).

Post hoc analysis using adjusted standardized residuals revealed an association between the differences retrieved and the ward of admission. *C. albicans* was reported more frequently in DACC when compared with other settings.

**Figure 4:**
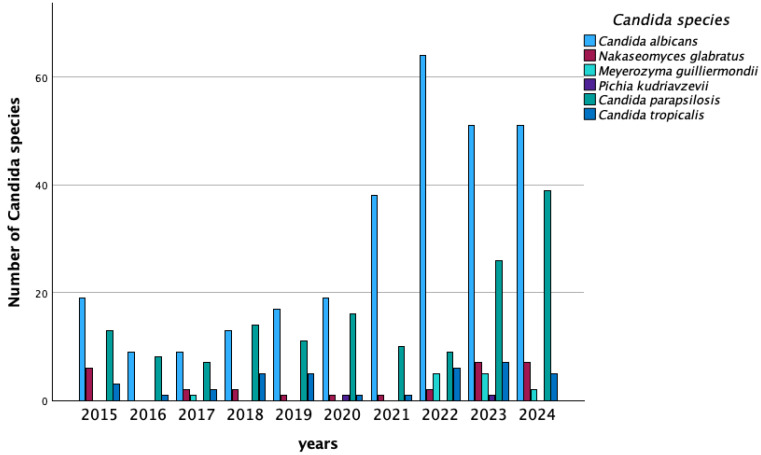
*Candida* species per year.

Between 2018 and 2024, 15 patients experienced sequential infections caused by two different *Candida* species. *C. albicans* was isolated in all these cases. The most frequent co-infection was with *C. parapsilosis* (8/15, 53%). The remaining cases were coinfected with *Meyerozyma guilliermondii* (*M. guilliermondii*) (4/15, 27%) and *C. tropicalis* (3/15, 20%). Notably, in 2022, an 82-year-old patient hospitalized in a medical unit experienced three subsequent episodes of Candidemia sustained by *C. albicans*, *C. parapsilosis* and *N. glabratus*, respectively. An upward trend in *C. albicans* was recorded over the period 2015–2024, with statistical relevance ^2^(9, 487) = 30.9, *p* < 0.001 (asymptotic 2-sided significance *p* < 0.001 at Pearson Chi-square). The temporal trend of candidiasis is represented in Figure 4.

### 4.3. Antifungal Resistance Profiles

During the first eight years of the study, the overall resistance rate of *C. albicans* to fluconazole increased from 16% (3/19) in 2015 to 19% (12/62) in 2022, followed by a decline in 2024 (6/51 resistant strains, 12% of total). Moreover, the resistance to voriconazole emerged, reaching 12% (6/51) in 2024. Similarly, resistance to amphotericin B of *C. albicans* increased from 10% (2/19) in 2015 to 21% (13/62) in 2022, but no resistant strains were reported in 2024. A key limitation of this study is the inability to confirm amphotericin B resistance by the reference broth microdilution method described by CLSI or EUCAST, or other commercial methods, which precludes definitive validation of the elevated resistance observed in 2022.

Over the same timeframe, the resistance rate of *N. glabratus* isolates to caspofungin rose from 1/6 cases in 2015 to 2/2 cases in 2022, with a reported turnaround of zero resistant strains during the latter two years of analysis. Since no EUCAST or CLSI clinical breakpoints are available for *V. (N.) glabratus*, and because MIC values in mg/L were not available from the commercial testing system used, it was not possible to apply EUCAST ECOFFs to determine wild-type status. Therefore, no conclusions regarding susceptibility to voriconazole or amphotericin B can be drawn.

The overall susceptibility rate of *C. parapsilosis* isolates to antifungals decreased over time. Resistance to fluconazole reached 13% (5/39), resistance to micafungin rose to 5% (2/39) and resistance to voriconazole increased to 13% (5/39). (Table 5).

## 5. Discussion

In this study, we analyzed the prevalence of candidemia in patients admitted to our University Hospital over 9 years. We observed an overall increase in cases sustained by *C. albicans* reporting resistance to both azoles and echinocandins. This finding has important implications for the empirical treatment of BSIs, particularly for patients reporting a high risk for candidemia, such as those with hematologic malignancies or receiving immunosuppressive therapies. The enrolled patients were predominantly elderly males hospitalized in Medical Units. This demographic profile differs from previous studies, which often focused on neutropenic patients or those admitted to ICUs, and represents a distinctive aspect of our cohort. It highlights the importance of considering new risk factors for *Candida*, such as total parenteral nutrition, extensive use of steroids, blood transfusion, and broad-spectrum antibiotics, with regard to the etiologic diagnosis of BSIs in medical wards. This is particularly relevant for patients experiencing prolonged hospitalization because of chronic diseases such as diabetes mellitus or chronic liver disease [4].

Consistent with existing literature, *C. albicans* was the leading cause of candidemia in our cohort [21]. Among *C.* non-*albicans* species, *C. parapsilosis* and *C. tropicalis* were the most prevalent. Over the study period, we observed an increasing trend of BSIs caused by *C. albicans* in medical units, while infections sustained by *C.* non-*albicans* species showed a decreasing trend. This pattern differs from that reported in other studies and may reflect the characteristics of our study population with a relatively high prevalence of aging patients [22].

In this context, Soriano Martín et al., in their 14-year surveillance study, reported that patients with *C. parapsilosis* candidemia were younger compared to those infected with *C. albicans* [23].

According to current clinical guidelines, echinocandins are recommended as first-line therapy for candidemia [10]; however, fluconazole remains an appropriate initial therapy in selected patients, particularly those who are not critically ill and have no prior exposure to azoles [6]. Consistent with literature, we noted a progressive increase in *C. albicans* resistance to azole during the first seven years of our study, mainly secondary to alteration of the target enzyme (cytochrome P-450 lanosterol 14 α-demethylase) mediated by the ERG11 gene; and failure of azoles to accumulate inside the fungi, followed by enhanced drug efflux mediated by Multidrug resistance (MDR) and Candida drug resistance (CDR) genes [24].

This trend could be explained by prior exposure, as this class of antifungals had been commonly used in the past as prophylaxis in immunocompromised patients, such as those with pharmacologically induced neutropenia or HIV infection with low CD4+ lymphocyte count and we could aspect some novelty in the next future due to the changes in prophylactic strategies in some populations at high risk for invasive fungal infections [25,26]. Interestingly, in 2023, voriconazole-resistant *C. parapsilosis* isolates (*n* = 3) outnumbered fluconazole-resistant isolates (*n* = 1). This finding may reflect a combination of factors: (i) differential selective pressure due to variations in the clinical use of voriconazole versus fluconazole; (ii) distinct resistance mechanisms, including specific ERG11 mutations or upregulation of efflux pumps (MDR, CDR), which may preferentially confer resistance to one azole; and (iii) incomplete cross-resistance between azoles (10.1093/ofid/ofac605).

**Figure 5:**
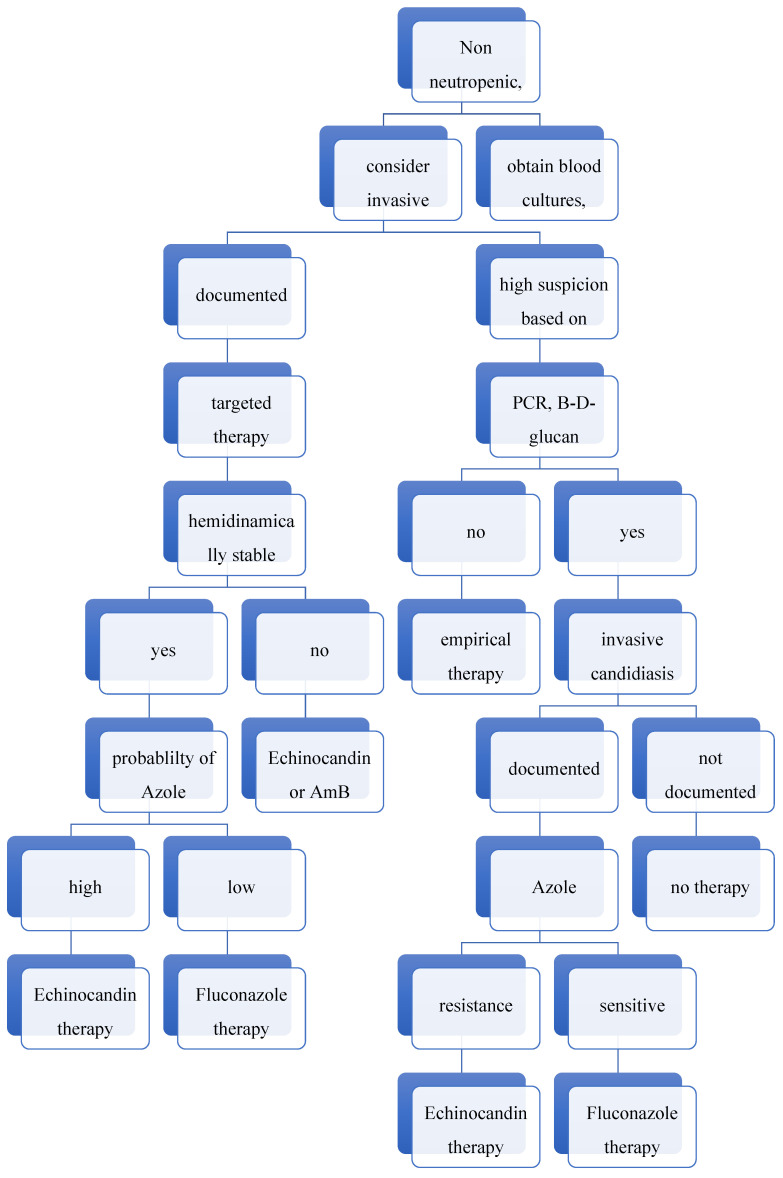
A therapeutic algorithmic approach for the management of invasive candidiasis.

As reported by Odoj et al. [27], more investments and innovation are needed to address gaps in antimycotic resistance, while a decrease in unnecessary antifungal prescription appears mandatory to decrease selective pressures [28]. A therapeutic algorithmic approach for the management of invasive candidiasis—generated, tested and implemented according to recent literature and our own local epidemiologic data—is reported in Figure 5.

Our study also highlights a concerning increase in resistance to echinocandins, particularly among *C.* non-*albicans* species. As expected, *C. parapsilosis* had a progressive decline in susceptibility to echinocandins over the study period, which may be attributed to an echinocandin overuse [29]. This phenomenon, also called “echinocandin heteroresistance”, describes a phenotypically resistant fungal subpopulation that could actively proliferate at a high concentration, potentially leading to treatment inefficacy. Only the phenotypically resistant cells survived during echinocandin treatment, causing systemic infection because of replication and translocation to deeper tissues. Notably, the increased virulence and pathogenicity of *C.* non-*albicans* species have been associated with significant morbidity and mortality, underscoring the need for continuous surveillance and targeted antifungal stewardship interventions [3].

Fungal infections pose a growing global health threat, especially as resistance to existing antifungal drugs increases. Developing new treatments is challenging due to the complex nature of fungal cells, while climate change and rising numbers of immunocompromised individuals further heighten the risk of widespread fungal disease. Vaccines offer a promising preventive strategy, with mRNA-based vaccines standing out after their recent success in other infectious diseases. By encoding fungal surface proteins, these vaccines could stimulate protective immune responses. However, their effectiveness will depend on identifying highly immunogenic fungal targets that are safe for humans. The urgent need for antifungal vaccines, particularly mRNA-based approaches, to combat rising antifungal resistance has been raised by Kumar and colleagues [30].

The primary limitation of our study is the absence of clinical data on prescribed antifungal therapy and patient follow-up, stemming from the unavailability of historical records.

In conclusion, our study provides insights into the ongoing epidemiological trends of BSIs sustained by *Candida*, highlighting the critical importance of implementing multidisciplinary antifungal stewardship programs to refine individualized treatment approaches and curb the rise in resistant strains. After investigating the characteristics of *Candida* spp. bacteremia in a large patient population, primarily composed of non-neutropenic individuals hospitalized in Medical Units, we found an increasing resistance rate to antifungals, including fluconazole and other azoles and echinocandins, likely reflecting the widespread use of these agents for both treatment and antifungal prophylaxis in particular settings such as OCUs and selected patients (i.e., those receiving long-term antibiotic therapies or parenteral nutrition). To address this growing challenge, the establishment of an antifungal management team, comprising physicians from various specialties (medical, surgery, and intensive care units), microbiologists and pharmacists, is essential to optimize antifungal stewardship, improve treatment and prophylaxis strategies, and ultimately curb the emergence of resistant *Candida* species.

## Figures and Tables

**Table 1:** Patient’s characteristics.

**Patients enrolled**	364	
**Number of strains**	487	
**Period of study**	1st January 2015–31st December 2024	
**Annual distribution of cases**	2015	27
	2016	15
	2017	28
	2018	32
	2019	33
	2020	29
	2021	49
	2022	84
	2023	68
	2024	72
**Gender, Male**	265 (54.4%)	
**Age (median (IQR))**	71 (17)	

**Table 2:** Hospitalization Wards.

Hospitalization Ward	Frequency/Strains, %	Age Median (IQR)
**Medical Units**	290 (59.5%)	76 (17)
**Surgery Units**	91 (18.7%)	69 (18)
**DACC**	77 (15.8%)	64 (24)
**Intensive Care Units**	29 (6%)	71 (10)

**Table 3:** Distribution of *Candida* spp. isolates (frequency and percentage).

*Candida* spp.	Frequency/Strains, %	Frequency/Strains, %
***C. albicans***	282 (57.9%)	
***C.* non-*albicans***	205 (42.1%)	
	*C. parapsilosis*	128 (26.3%)
	*C. tropicalis*	35 (7.2%)
	*N. glabratus*	28 (5.7%)
	*M. guilliermondii*	12 (2.5%)
	*Pichia kudriavzevii*	2 (0.4%)
	Total	487 (100%)

**Table 4:** Relation between *Candida* species strains and hospital wards.

			*C. albicans*	*C.* non-*albicans*	Total
**Hospital Units**	**Surgery**	Count	32	59	91
		% within a kind of unit	35.2%	64.8%	100%
		% within the kind of *Candida* spp.	15.6%	20.9%	18.7%
	**Medical Units**	Count	135	155	290
		% within kind of unit	46.6%	53.4%	100%
		% within the kind of *Candida* spp.	65.9%	55%	59.5%
	**DACC**	count	24	53	77
		% within a kind of unit	31.2%	68.8%	100%
		% within the kind of *Candida* spp.	11.7%	18.8%	15.8%
	**Intensive Care Unit**	count	14	15	29
		% within a kind of unit	48.3%	51.7%	100%
		% within the kind of *Candida* spp.	6.8%	5.3%	6%
**total**		count	205	282	487
		% within a kind of unit	42.1%	57.9%	100%
		% within the kind of *Candida* spp.	100%	100%	100%
		**Value**	**Df**	**Asymptotic significance (2-sided)**	
**Pearson chi-square**		8.382	3	0.039 (<0.05)	
**Likelihood ratio**		8.518	3	0.036	
**N of valid cases**		487			

**Table 5:** Resistance profile of *Candida* spp. through the years.

Species (*n*)	Agent	Year									
		2015	2016	2017	2018	2019	2020	2021	2022	2023	2024
***C. albicans* (*n*)**		19	9	14	13	17	19	38	62	51	51
	Amph B	2	1	1	1	2	2	3	13	2	0
	Caspofungin	1	0	2	0	1	1	1	6	4	2
	Fluconazole	3	1	0	0	1	1	3	12	9	6
	Micafungin	2	0	0	1	2	1	1	7	6	3
	Voriconazole	0	0	2	0	1	2	2	8	5	6
***C. parapsilosis* (*n*)**		13	8	7	14	11	16	10	10	26	39
	Amph B	0	0	0	0	0	1	0	2	0	0
	Caspofungin	0	0	0	0	0	0	0	1	0	0
	Fluconazole	0	1	0	1	4	1	0	1	1	5
	Micafungin	0	0	0	0	0	0	0	3	0	2
	Voriconazole	0	1	0	1	4	0	0	3	0	5
***N. glabratus* (*n*)**		6	0	4	2	1	1	1	2	7	7
	Amph B	0	0	0	0	0	0	0	0	1	0
	Caspofungin	1	0	1	0	1	1	0	2	2	1
	Fluconazole	0	0	0	0	0	0	0	0	0	0
	Micafungin	1	0	1	0	1	0	0	1	0	0
	Voriconazole	0	0	0	0	0	0	0	0	0	0
***C. tropicalis* (*n*)**		3	1	3	5	5	1	1	6	7	5
	Amph B	0	0	0	0	0	0	0	0	0	0
	Caspofungin	0	0	0	0	0	0	0	0	0	0
	Fluconazole	0	0	0	2	0	0	0	0	0	1
	Micafungin	0	0	0	2	0	0	0	I	0	0
	Voriconazole	0	0	0	0	0	0	0	0	0	0

## Data Availability

The data presented in this study are available upon request from the corresponding author.
